# Effective elimination of bacteria on hard surfaces by the combined use of bacteriophages and chemical disinfectants

**DOI:** 10.1128/spectrum.03797-23

**Published:** 2024-03-14

**Authors:** Zongyue Chen, Yuhui Yang, Gaoming Li, Youying Huang, Yu Luo, Shuai Le

**Affiliations:** 1 School of Nursing, Army Medical University, Chongqing, China; 2 Disease Surveillance Division, Center for Disease Control and Prevention of Central Theater Command, Shijingshan, Beijing, China; 3 Biomedical Analysis Center, College of Basic Medical Sciences, Army Medical University, Chongqing, China; 4 Department of Microbiology, College of Basic Medical Sciences, Key Laboratory of Microbial Engineering Under the Educational Committee in Chongqing, Army Medical University, Chongqing, China; Centre de Biologie Integrative, Toulouse, France

**Keywords:** phage, chemical disinfectant, clinical environmental disinfection

## Abstract

Hospital-acquired infections (HAIs) represent one of the significant causes of morbidity and mortality worldwide, and controlling pathogens in the hospital environment is of great importance. Currently, the standard disinfection method in the hospital environment is chemical disinfection. However, disinfectants are usually not used strictly according to the label, making them less effective in disinfection. Therefore, there is an emergent need to find a better approach that can be used in hospitals to control pathogenic bacteria in the clinical environment. Bacteriophages (phages) are effective in killing bacteria and have been applied in the treatment of bacterial infections but have not received enough attention regarding the control of contamination in the clinical environment. In this study, we found that various phages remain active in the presence of chemical disinfectants. Moreover, the combined use of specific phages and chemical disinfectants is more effective in removing bacterial biofilms and eliminating bacteria on hard surfaces. Thus, this proof-of-concept study indicates that adding phages directly to chemical disinfectants might be an effective and economical approach to enhance clinical environment disinfection.

**IMPORTANCE:**

In this study, we investigated whether the combination of bacteriophages and chemical disinfectants can enhance the efficacy of reducing bacterial contamination on hard surfaces in the clinical setting. We found that specific phages are active in chemical disinfectants and that the combined use of phages and chemical disinfectants was highly effective in reducing bacterial presence on hard surfaces. As a proof-of-concept, we demonstrated that adding specific phages directly to chemical disinfectants is an effective and cost-efficient strategy for clinical environment disinfection.

## INTRODUCTION

In recent years, hospital-acquired infections (HAIs) have emerged as a significant public health concern worldwide, attracting increased attention (1, 2). HAIs have led to numerous severe and even fatal cases (3). Hospital environmental contamination plays a pivotal role in increasing the risk of HAIs. Therefore, effective disinfection of hospital environmental surfaces is highly crucial in reducing the risk of HAIs. Traditional disinfection methods, such as ultraviolet-C (UV-C) or chemical disinfection, are no longer sufficient to address the challenges posed by drug-resistant bacteria in hospital environments (4). Therefore, it is necessary to explore innovative disinfection methods that can handle the limitations of traditional approaches, while consistently and effectively targeting multidrug-resistant (MDR) bacteria in a hospital environment.

Bacteriophages are viruses that specifically infect bacteria. Initially, they were recognized for their potential as natural bactericidal agents (5, 6). However, research on phages focuses primarily on their application in clinical infection treatment, with limited exploration of their use in environmental disinfection (7–10). While some studies have demonstrated successful applications of phages in food processing plants, livestock farms, and fields, the research on phages in clinical environment disinfection is limited (11–15).

In this study, we conducted further investigations on phage-based strategies in combination with chemical disinfectants to maximize their potential for disinfecting clinical environments. This proof-of-concept study indicates that adding phages to chemical disinfectants might enhance the efficiency of clinical environment disinfection.

## RESULTS

### The effects of chemical disinfectants on phages

First, we tested whether chemical disinfectants led to inactivation of the phages, and the sensitivity of the eight phages against four different disinfectants was assessed (Tables 1 and 2). The tested concentrations of disinfection are those commonly employed in hospital environments (16–22). Among the eight tested phages, seven are dsDNA phages with the protein capsid, and phiYY is a dsRNA phage belonging to Cystoviridae. Its capsid is encapsulated by an external lipid envelope (23, 24).

**TABLE 1:** Four different types of chemical disinfectants

Disinfectant	Sodium dichloroisocyanurate	Benzalkonium chloride	Hydrogen peroxide	Chlorhexidine digluconate
Chemical structure	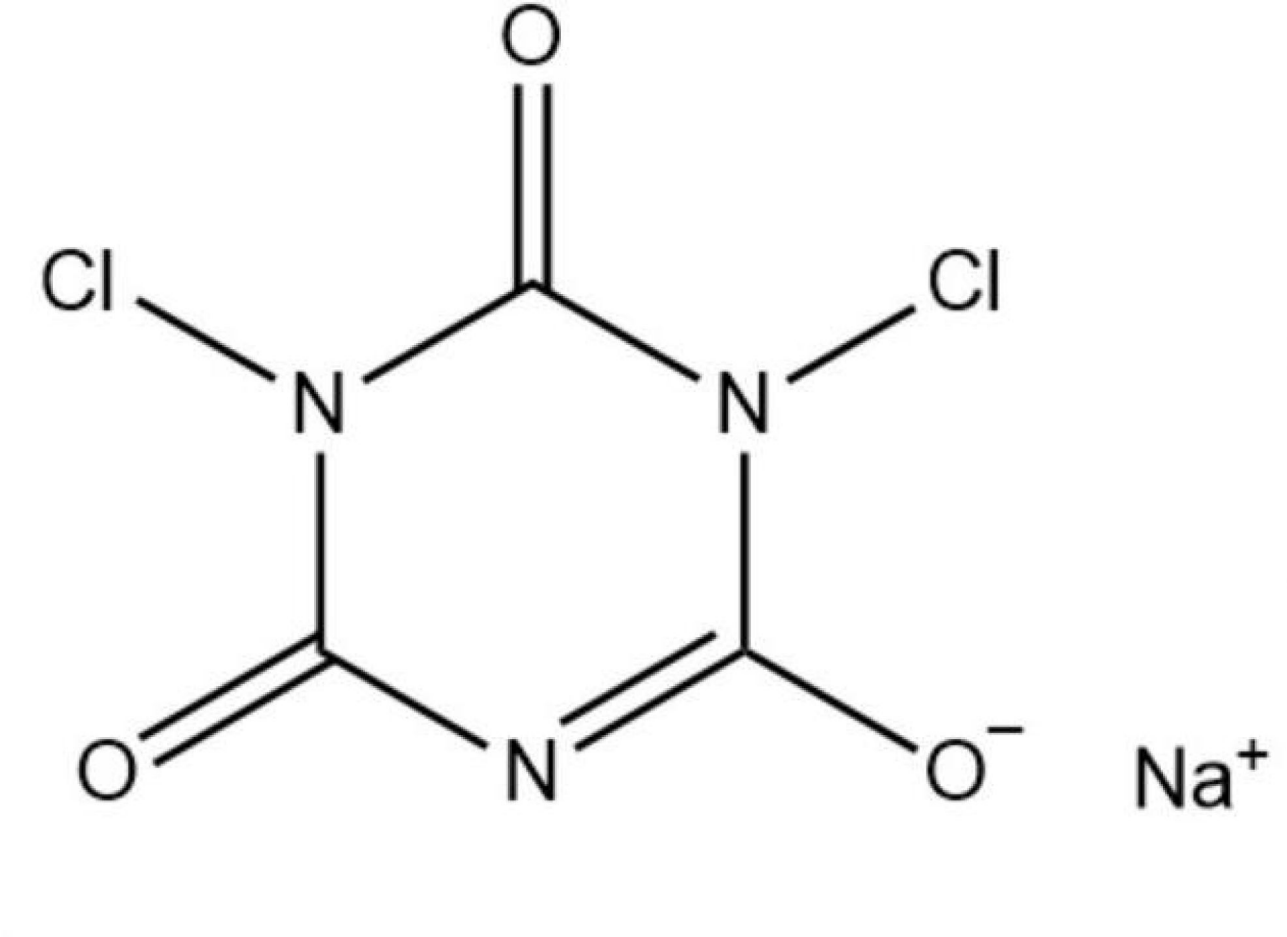	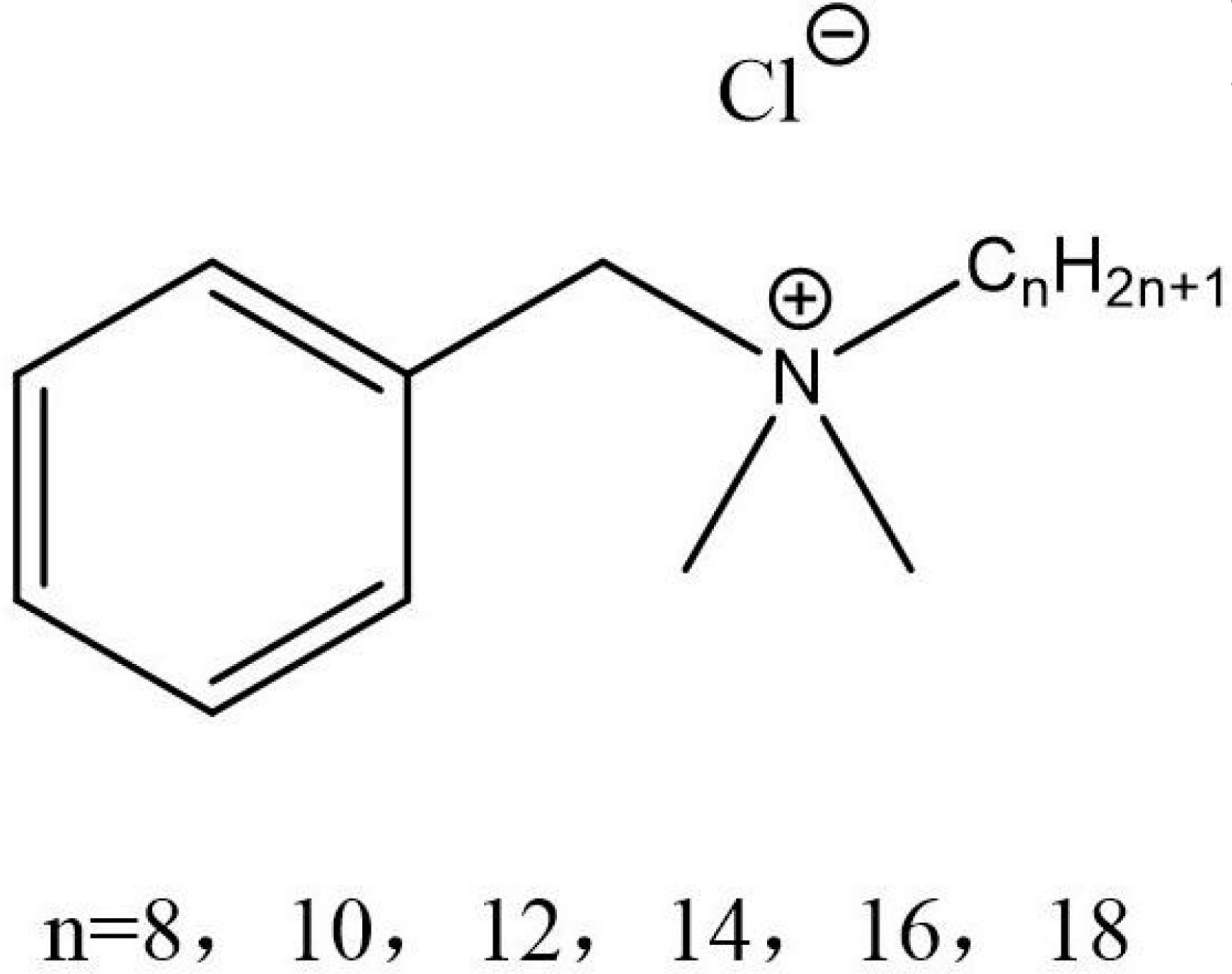	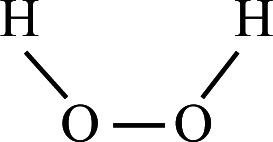	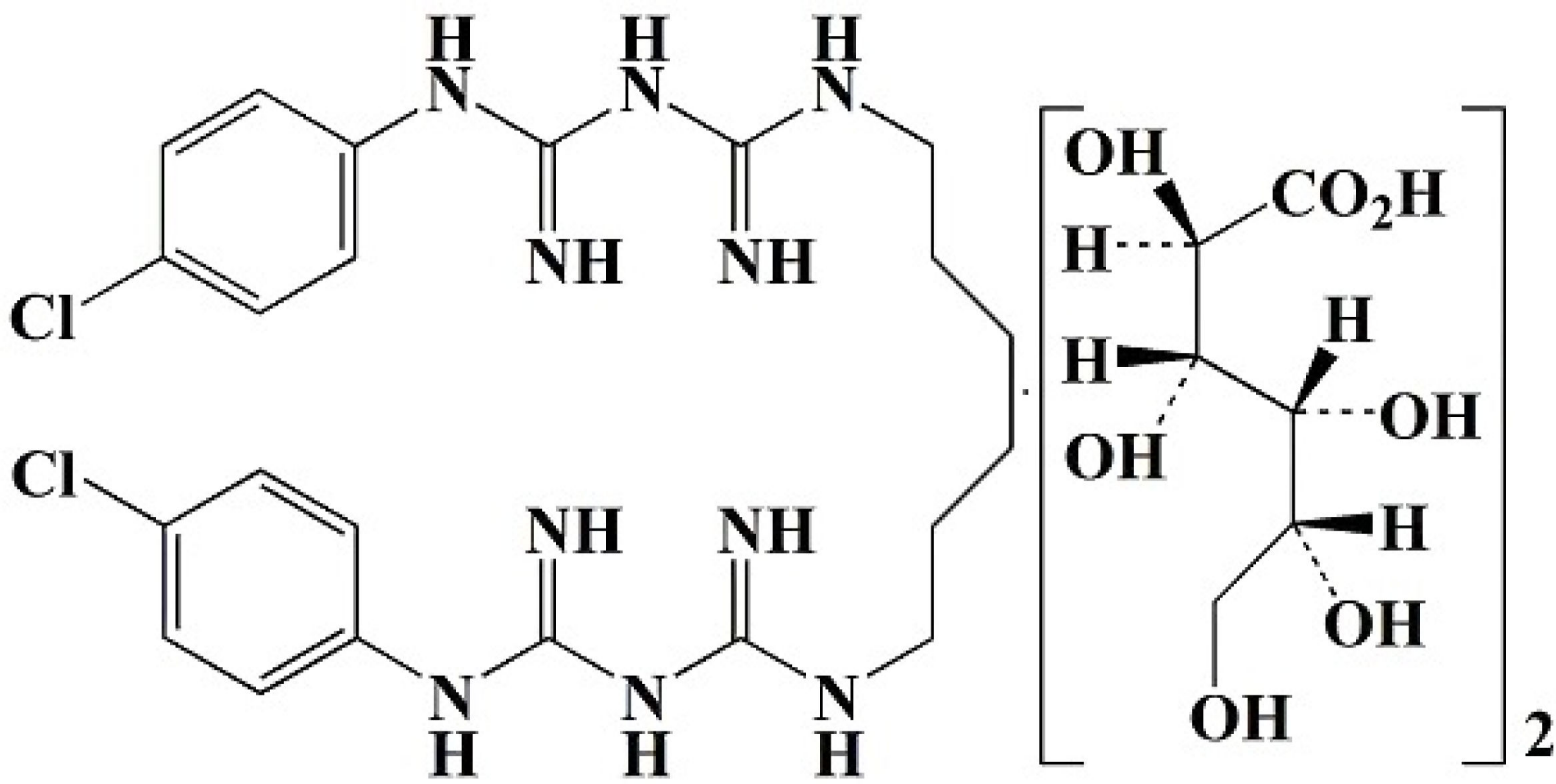
Chemical class	Phenolic compound	Quaternarium ammonum compound (QAC)	Oxidizing agent	Biguanide
Mode of action	Membrane disruption	Membrane damage	Oxidative damage	Membrane damage
Final concentration	0.05% (500 mg/L)	0.5% (5000 mg/L)	3% (30,000 mg/L)	0.5% (5000 mg/L)
Reference	(19)	(20)	(22)	(21)

**TABLE 2:** Selected strains and phages in this study

Strain	Description	Reference
Ab9	Wild-type *Acinetobacater baumannii* strain	(25)
THR60	Wild-type *Klebsiella pneumoniae* strain	This study
JM110	Mutant type *Escherichia coli* strain	(26)
PAO1	Wild-type *Pseudomonas aeruginosa* strain	(27)
PAO1r	*P. aeruginosa* strain with a rough LPS phenotype	(28)
Abp9	dsDNA *A. baumannii* bacteriophage	(29)
GZ7	dsDNA *K. pneumoniae* bacteriophage	This study
T5	dsDNA *E. coli* bacteriophage	(30)
phiYY	dsRNA *P. aeruginosa* bacteriophage	(23)
PaoP5	dsDNA *P. aeruginosa* bacteriophage	(24)
PaP16-a	dsDNA *P. aeruginosa* bacteriophage	This study
PaP21-1	dsDNA *P. aeruginosa* bacteriophage	This study
PaP24-X	dsDNA *P. aeruginosa* bacteriophage	This study

**Fig 1:**
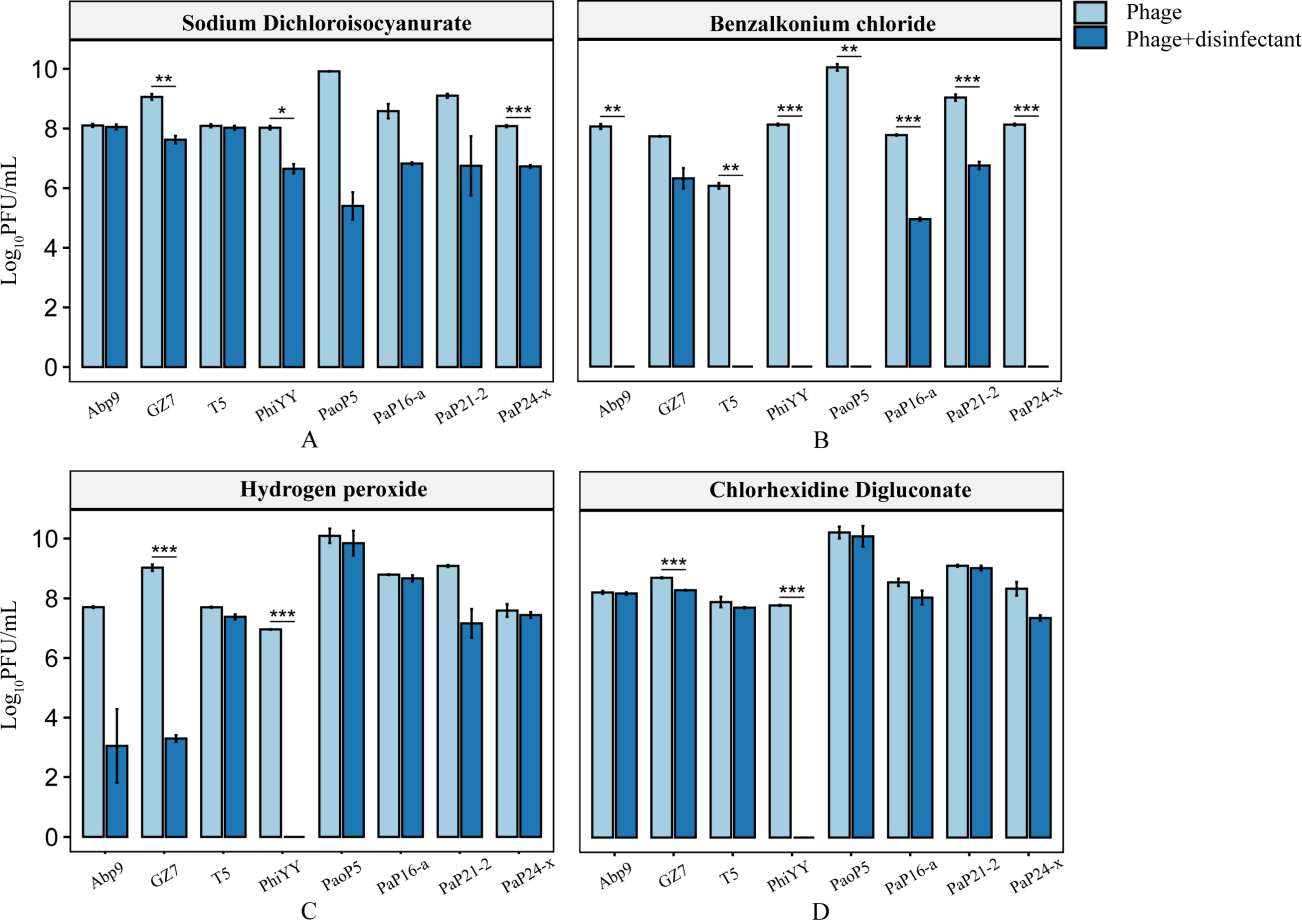
The impact of (**A**) Sodium dichloroisocyanurate (0.05% wt/vol), (**B**) benzalkonium chloride (0. 5% wt/vol), (**C**) hydrogen peroxide (3% wt/vol), and (**D**) chlorhexidine digluconate (0.5% wt/vol) on phages. The data represent the mean of three biological replicates, and the phage titer is expressed in logarithmic form. Chemical disinfectants have different effects on phages. "Phage +disinfectant" stands for the experimental group, and "Phage" stands for the control group. All phages were incubated with chemical disinfectants at 37°C for 1 hour, and then the remaining viable phages were determined by plaque assays. The results showed some phages that could survive in the disinfectants. The experiment includes three biological replicates. The data are represented as means ± SD. *P < 0.05, **P < 0.01, and ***P < 0.001, determined by independent *t*-test.

First, the phages were exposed to the disinfectants for 1 hour, and then the remaining viable phages were determined by plaque assays. Among the four chemical disinfectants, benzalkonium chloride (0.5% wt/vol) was very effective in killing the phages, in which most phages (e.g., Abp9, T5, and phiYY) were completely inactivated to undetectable levels (Fig. 1B). However, the other three chemical disinfectants, which are commonly applied in hospital environment disinfection, had a minimal impact on the titers of the phages, except phage phiYY, which was extremely sensitive to all the disinfectants due to the presence of the lipid envelope (23) (Fig. 1).

Sodium dichloroisocyanurate (0.05% wt/vol) was not adequate for inactivating the phages, which only reduced the titers of some phages (e.g., phiYY and PaoP5) by two to four orders of magnitude (Fig. 1A). Some of the tested phages, such as GZ7, PaP16-a, and PaP21-2, were more resistant to the four types of chemical disinfectants than other phages, which were not inactivated by the four disinfectants (Fig. 1). Thus, we were able to identify specific phages that could survive in the chemical disinfectants.

### Phage and chemical disinfectants remove biofilms efficiently

**Fig 2:**
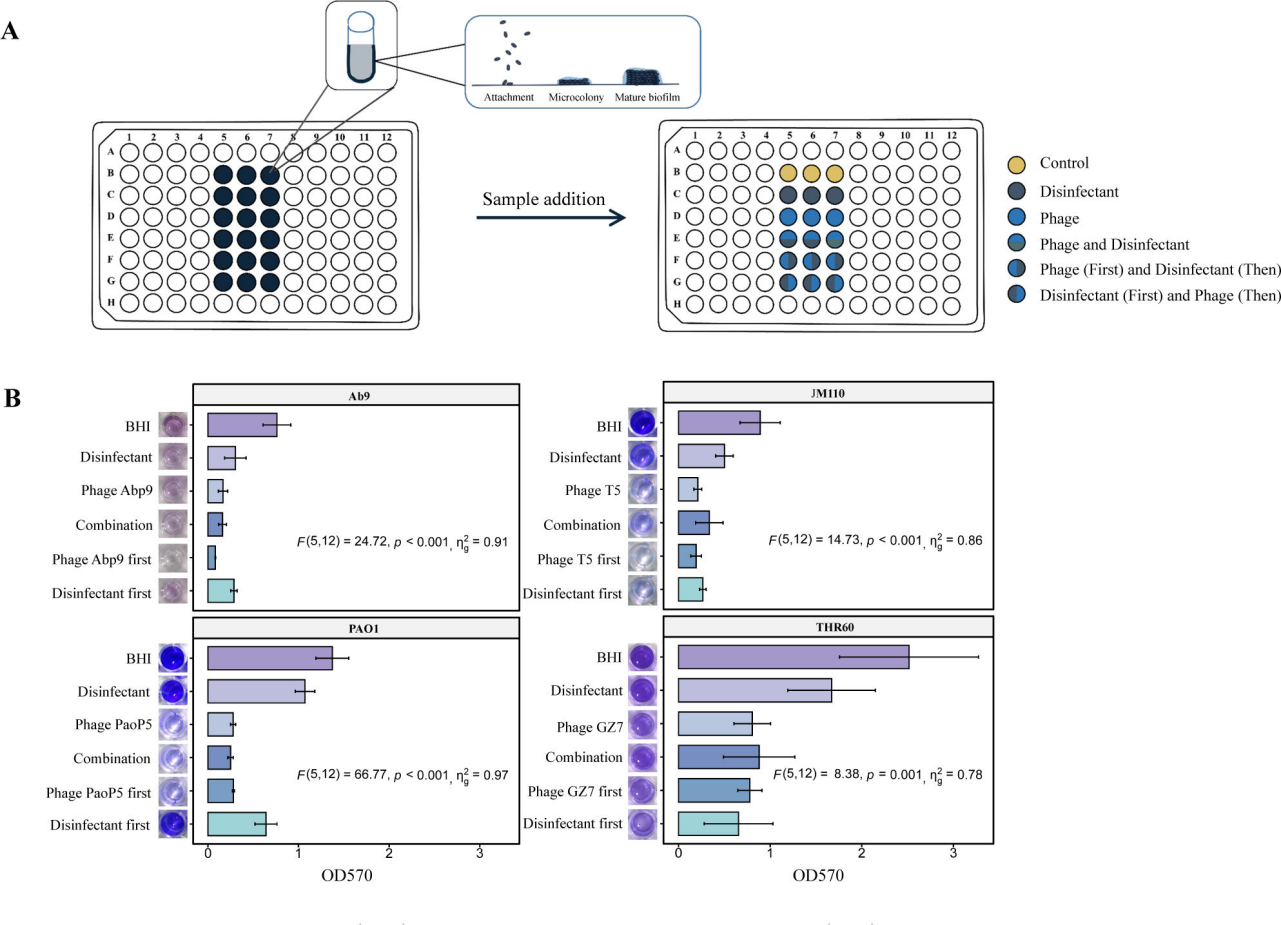
(**A**) Schematic diagram of the biofilm removal experiment. Yellow represents brain heart infusion (BHI). Dark green represents sodium dichloroisocyanurate (0.05% wt/vol). Blue represents phages. Blue above and dark green below represent the simultaneous treatment of bacteria with phage and sodium dichloroisocyanurate, respectively (0.05% wt/vol). Blue on the left and dark green on the right indicate that the phages were applied first, and sodium dichloroisocyanurate (0.05% wt/vol) was used for treatment afterward. Dark green on the left and blue on the right indicate that sodium dichloroisocyanurate (0.05% wt/vol) was used for treatment first, and phages were applied afterward. (**B**) Results of biofilm clearance experiments. The data represent the mean of three biological replicates, the six graphs on the left of each group show the results of biofilm crystal violet staining, and the bar graph on the right represents the optical density at 570 nm (OD_570_) values. "Disinfectant" represents sodium dichloroisocyanurate (0.05% wt/vol); "Phage" represents Abp9, T5, PaoP5, or GZ7; "Combination" represents phages and sodium dichloroisocyanurate (0.05% wt/vol); "Phage first" represents the phage used first and then sodium dichloroisocyanurate (0.05% wt/vol); "Disinfectant first" represents sodium dichloroisocyanurate (0.05% wt/vol) used first and then phages. The data were presented as means ± SD. Statistical analysis was conducted using one-way ANOVA. The effect sizes (η_g_^2^) demonstrated the relative magnitude of strain differences observed in each experiment. Inferential statistical comparison resulted in suggestive evidence (*P* < 0.05).

The biofilm is an important source of bacterial contamination on hospital environmental surfaces, and its unique physiological and structural characteristics make it resistant to chemical disinfectants (31). Four types of pathogenic bacteria and their phages were chosen to examine the potential interactions between phage and chemical disinfection for biofilm removal. These strains include *A. baumannii* Ab9, *K. pneumoniae* THR60, *E. coli* JM110, and *P. aeruginosa* PAO1. Correspondingly, the phages used were Abp9, GZ7, T5, and PaoP5 (Table 2). Then, 24-h biofilms of the four strains were treated with different combinations of phages at a final titer of 10^10^ PFU/mL and sodium dichloroisocyanurate (0.05% wt/vol) (Fig. 2A).

The biofilm removal rates of *K. pneumoniae* THR60 and *E. coli* JM110 were 68% and 76% with phage treatment alone and 65% and 62% with the combination treatment, respectively. The results indicated that the combination treatment was not consistently superior to that with phages. The possible reasons for this discrepancy may include a potential decrease in the lytic activity of the phages in the presence of sodium dichloroisocyanurate or the alteration of bacterial states caused by sodium dichloroisocyanurate, which could impact phage propagation. Therefore, treatments were applied sequentially. The biofilms of *P. aeruginosa* PAO1 were removed more efficiently when phage treatment was applied first. The removal rate achieved was 80%, but it was less efficient when the disinfectant was used first [*F* (5, 12)=66.77, *P < 0.001*] (Table S1). These results suggested that combining phage with sodium dichloroisocyanurate may enhance the efficacy of disinfection.

### Surface decontamination by the phages and sodium dichloroisocyanurate

**Fig 3:**
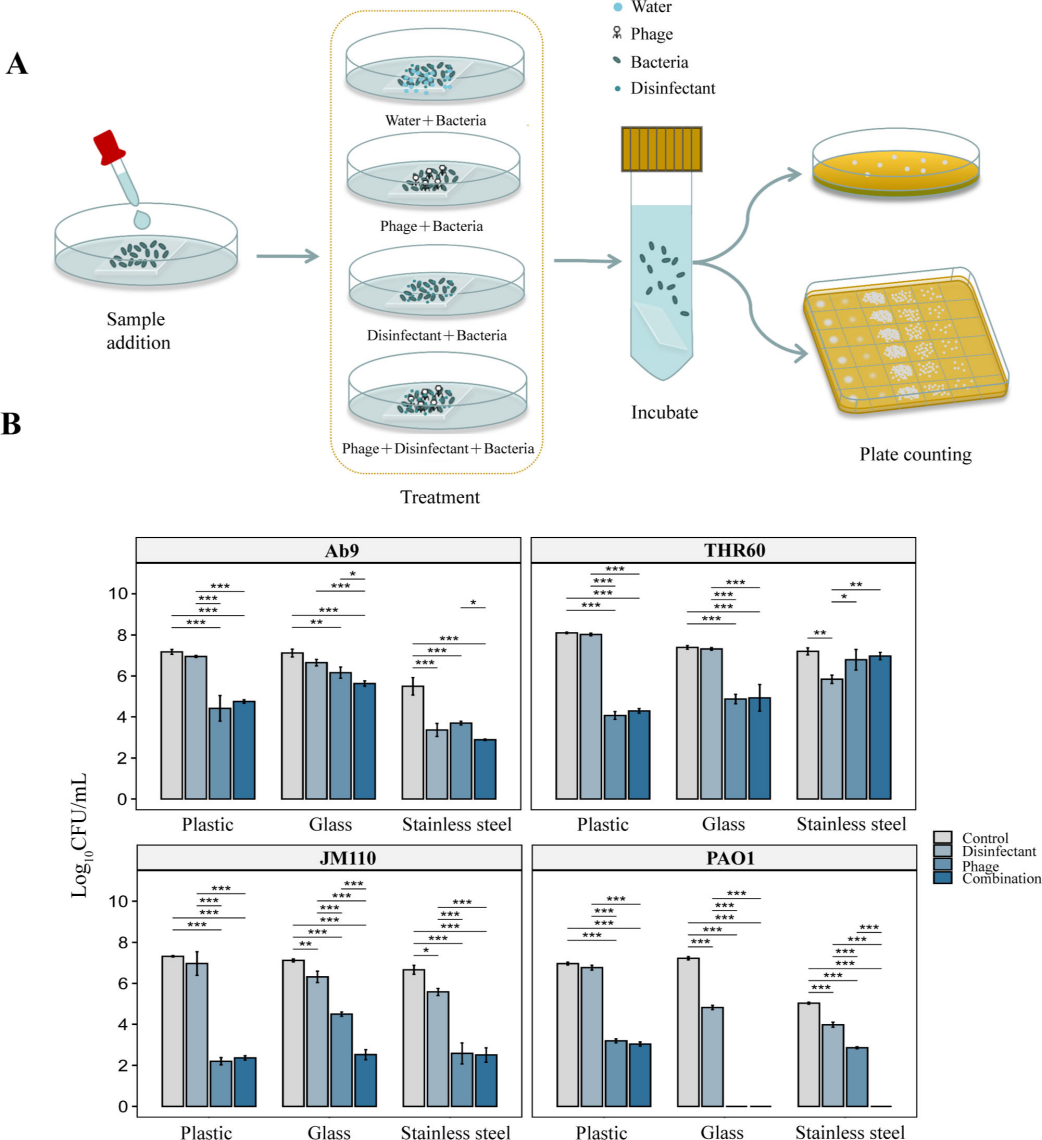
(**A**) Disinfection experiment on hard surfaces of the hospital environment. Sample addition: contaminate hospital environmental surfaces (plastic, glass, and stainless steel) with pathogens. Treatment: treat contaminated hard surfaces with different methods (phage and disinfectant). Incubation: collect bacteria. Count: colony counting. (**B**) Results of disinfection experiments with phage and sodium dichloroisocyanurate. Treatment with phages alone and the combination of phages with sodium dichloroisocyanurate were effective in reducing the bacterial load on hard surfaces. The data are shown as means ± SD. *P < 0.05, **P < 0.01, and ***P < 0.001, determined by one-way ANOVA.

Because the type of the surface material may affect the effectiveness of disinfection (32), as a proof-of-concept experiment, "surface decontamination" studies were performed on three small pieces of distinct material (plastic, glass, and stainless steel) to mimic the hard surfaces in a hospital environment. After contaminating the surface with specific pathogenic bacteria for 1 hour, the materials were treated with phages and sodium dichloroisocyanurate for 2 hours (Fig. 3A). Then, a standard plate count method was used to determine the remaining pathogenic bacteria on the surfaces. For plastics contaminated with *A. baumannii* Ab9, the colony forming units (CFUs) significantly reduced (three to four orders of magnitude) [*F* (3, 8)=60.42, *P < 0.001*] (Table S2）in the phage treatment group and phage–sodium dichloroisocyanurate treatment group when compared with the sodium dichloroisocyanurate treatment alone group. Stainless steel experimentally contaminated with *A. baumannii* Ab9 showed a statistically significant difference between the phage treatment group and phage–sodium dichloroisocyanurate treatment group *[F* (3, 8)=53.14, *P < 0.001*] (Fig. 3B; Table S2).

In addition, we observed notable variations in the disinfection efficacy of phages and sodium dichloroisocyanurate on experimentally contaminated surfaces, depending on the specific pathogens involved (Fig. 3B; Table S2). Specifically, the treatments led to a statistically significant effect in reducing the bacterial load on glass and stainless steel contaminated with *P. aeruginosa* PAO1 (*F* (3, 8)=2962.36, *P < 0.001*) (Fig. 3B; Table S2). When treated with phages alone or in combination with sodium dichloroisocyanurate, the *P. aeruginosa* PAO1 on glass and stainless steel was significantly reduced by six to seven orders of magnitude. Similarly, on plastic surfaces, the *P. aeruginosa* PAO1 was decreased by three to four orders of magnitude when treated with phages alone or in combination with sodium dichloroisocyanurate (Fig. 3B; Table S2). This highlights the potential effectiveness of phages in disinfecting environmental surfaces contaminated with *P. aeruginosa*.

All the studies have demonstrated that the effectiveness of different disinfection methods varies depending on the specific bacteria being targeted and the type of surface material. However, the overall trend observed indicates that both treatment with phages alone and with the combination of phages with sodium dichloroisocyanurate effectively reduced the bacterial load on the surfaces.

## DISCUSSION

The significance of HAIs has garnered substantial attention worldwide. In the United States, the Centers for Disease Control and Prevention (CDC) estimates that HAIs contribute to an estimated 99,000 deaths annually (33–35). The emergence of MDR bacteria renders traditional antibiotic therapies less effective in treating HAIs and exacerbates the severity of the issue (36). Extensive research has shown that the hospital environment can serve as a reservoir for pathogens responsible for HAIs (37–44). For example, *P. aeruginosa* can survive on hospital surfaces for up to 16 months, while *K. pneumoniae* can survive up to 30 months (45). On the contrary, disinfectants are usually not used strictly according to the label, making them less effective in disinfection (46).

For an extensive period, UV-C and chemical disinfectants have demonstrated promising disinfection efficacy in hospital environments. However, a multitude of concerns have also emerged concurrently. UV-C disinfection is limited in its application due to the presence of patients in the room, and the concentration of chemical disinfectants used must be carefully regulated to ensure both their effectiveness and safety (47–49). Moreover, the constant movement of personnel in hospitals facilitates rapid recolonization of environmental surfaces by bacterial pathogens, further complicating the task of disinfecting the ward environment (50–53). Studies have shown that repeated exposure to sub-inhibitory concentrations of a specific disinfectant may lead bacteria to develop tolerance to the disinfectant (54–59).

The search for alternative disinfectants should prioritize the efficacy, safety, and the ability to target a broad spectrum of drug-resistant pathogens. This will ensure the development of effective disinfection strategies to mitigate the risks associated with HAIs and enhance patient safety. Compared to chemical disinfectants, phages possess several advantages. First, phages represent a vast microbial resource with immense potential for various applications, targeting specific bacteria that may exhibit multiple antibiotic resistance or are prone to forming biofilms. Second, phages are viruses that exclusively infect bacteria and do not affect mammalian cells, making them safe for humans (60, 61). Consequently, using phages for thorough disinfection in hospital environments is considered safe (62, 63). Further research and exploration of phage-based disinfection strategies are needed.

Several studies have investigated the direct application of phages as disinfectants on surfaces in hospital environments, demonstrating their potential in eliminating pathogens effectively(27) (64–66). Additionally, phages have shown effectiveness in removing biofilms formed by various pathogenic bacteria, including *P. aeruginosa, A. baumannii,* and *K. pneumoniae* (67, 68). While these studies have provided evidence for the effectiveness of phages in reducing pathogenic microorganisms in hospital environments, there is limited research on the evaluation of combining phages with chemical disinfectants (64, 69). In this article, we focused on studying the effect of disinfectants on phages and discovered that specific phages can tolerate clinical disinfectants. More combination effects were also observed in biofilm removal and bacteria removal from hard surfaces.

### Phages lyse bacteria in the presence of chemical disinfectants

Phages will almost certainly encounter chemical disinfectants when they are used for clinical disinfection. Consequently, it is essential to develop phage-based products to assess the potential impact of chemical disinfectants on phages. Many other studies have explored the susceptibility of phages that inactivate bacterial pathogens to chemical disinfectants, with different results. The titers of two *E. coli* phages were significantly reduced after coincubation with 70% alcohol or chemical disinfectants containing octenidinum dihydrochloride and phenoxyethanol (65). An *S. aureus* phage was cocultivated with four chemical disinfectants, but only benzalkonium chloride and hydrogen peroxide impacted the phage’s activity.

In contrast, the other chemical disinfectants, trichloromethane and chlorhexidine, did not affect the phage’s activity (66). David *et al*. found that at least 10^2^–10^3^ PFU/mL of the initial number of phages survived in the presence of widely used traditional disinfectants applied daily in food environments (70). Our study showed that most phages exhibited high resistance to the tested disinfectants, further demonstrating the utility of phages as a disinfectant for bacteria in hospital environments.

In this study, benzalkonium chloride (0.5% wt/vol) had the most significant effect on the phages. However, results from other studies indicate phages are resistant to benzalkonium chloride. Stachler *et al*. reported that *P. aeruginosa* phages resisted benzalkonium chloride (69, 71). Similarly, benzalkonium chloride did not significantly affect the *Staphylococcus* phage at bacteriostatic concentrations (66). This difference may be due to the benzalkonium chloride concentrations that were used. In this study, we employed the clinical concentration of benzalkonium chloride. Conversely, the previous studies used a relatively low concentration of benzalkonium chloride, which might explain why they did not observe any significant impact on phages. The aforementioned results further illustrate that it is necessary to continue isolating phages that demonstrate resistance to chemical disinfectants if we intend to use them in clinical settings for disinfection. This also emphasizes the importance of identifying and isolating phages for their specific applications in clinical environment disinfection in the future.

### The combination of phage and chemical disinfectants can be more effective in removing biofilms

It is widely recognized that bacteria can form biofilms as a survival mechanism in hostile environments, thereby increasing their resistance to antibiotics or disinfectants (71, 72). Therefore, effective removal of biofilms present on surfaces within hospital environments is an essential component of clinical environment disinfection (73). Previous studies have consistently shown that phages were effective in eradicating bacteria within biofilms. Zhang *et al*. observed approximately 75% reduction in *P. aeruginosa* biofilms treated with phages for 72 hours. Another study found more than 99% biofilm removal for monophage treatments (69, 74). Comparatively, this study found that phage treatment alone for 2 hours resulted in approximately 74% biofilm removal. However, previous research has indicated that a low phage concentration not only failed to remove biofilms but could also stimulate biofilm growth (75). These contradictory results highlight the importance of phage selection and characterization for future phage-based formulations.

Fewer studies have explored the combinatory effect of phages and chemical disinfectants on biofilms (76, 77). Agún *et al*. found that combining phages with chlorhexidine cannot increase the removal of biofilms (66). On the contrary, the combination treatment with sodium hypochlorite and phages revealed additive effects, and biofilms were removed entirely (78). Stachler *et al*. reported that pre-treating *P. aeruginosa* biofilms with phages followed by treatment with chemical disinfectants proved effective in biofilm removal (69). Consistent with previous studies, our study found that for treating the biofilms formed by *A. baumannii* Ab9, *P. aeruginosa* PAO1, and *E. coli* JM110, the treatment of biofilms with phages followed by treatment with sodium dichloroisocyanurate resulted in more significant removal of biofilms than the treatment with sodium dichloroisocyanurate followed by phage treatment. This phenomenon may be caused by the change in the state of the bacteria after pre-treatment with chemical disinfectants. Applying chemical disinfectants may affect the physiology of the bacteria, which might affect phage replications (79).

### The combination of phages and chemical disinfectants can effectively reduce the pathogenic bacteria on the hard surfaces of hospital environments

Previous studies have investigated the impact of chemical disinfectants or phages for decontaminating bacteria-contaminated hard surfaces in hospital environments (80, 81). A *Salmonella* phage mixture could effectively reduce *Salmonella Kentucky* on glass and steel surfaces (80). Similarly, Viazis *et al*. found that the phage mixture BEC8 could reduce *E. coli* on stainless steel and ceramic surfaces (80). Some phages could decontaminate all types of hard surfaces tested (e.g., glass, plastic, and ceramic) without any significant difference between surface types or bacterial strains (82). Our study found that phages could effectively reduce bacterial load on hard surfaces. Phage PaoP5 effectively reduced the bacterial load on all three hard surfaces tested. This further demonstrated the utility of phages as a treatment for bacteria on hospital surfaces.

To date, fewer studies have examined the effectiveness of phage–disinfectant combinations in decontaminating hard surfaces. Stachler *et al.* reported that phage treatment before chemical disinfection can enhance the removal of plastic-surface-associated *P. aeruginosa* (69). In the current study, the combined use of phage and chemical disinfectants significantly reduced pathogenic bacteria on hard surfaces. Considering that phages are safe for humans and even used in therapy, combining phages and disinfectants could be regarded as a preventive control strategy. This approach can help prevent recurrent infections and combat outbreaks caused by specific pathogens in particular environments.

### Limitations of the study and future research directions

This is a proof-of-concept study with several limitations. First, this study was conducted under laboratory conditions, which may not fully replicate the complexity and variability of natural hospital environments. The study did not investigate the impact of other factors, such as pathogen load, environmental temperature, or nutrient abundance. However, this study is a proof-of-concept experiment indicating that specific phages can survive in disinfectants and enhance elimination efficacy, which provides valuable data to support further research on related topics in hospital environments (83).

Second, this study focused on a limited number of phages and pathogenic bacteria, and the microbial composition on surfaces in natural clinical environments may be more diverse and complex. Therefore, future studies should consider the microbial diversity on clinical surfaces and investigate whether the presence of non-host bacteria could affect the lytic activity of phages against host bacteria. Moreover, the study focused on a limited number of phages and chemical disinfectants, and there may be other phage–chemical disinfectant combinations worth exploring.

Lastly, considering the strict host specificity of phages, their application for targeted disinfection in clinical settings requires the isolation of specific phages and a thorough evaluation of their interactions with chemical disinfectants before use (84–86). Therefore, future research efforts should focus on developing effective and efficient methods for isolating phages resistant to chemical disinfectants. A phage cocktail comprising multiple phages could offer a more targeted solution, and further investigation of the combination with a phage cocktail is also essential (87). Moreover, synthetic biology technology might be applied to expand the host ranges of phages and enhance tolerance of phage to disinfectants, which deserves further investigation (88, 89).

In summary, this study demonstrates the feasibility and potential of employing phages and chemical disinfectants for surface disinfection in hospital environments.

## MATERIALS AND METHODS

### Bacterial strains, phages, culture conditions, and disinfectant formulations

This study used four types of pathogenic bacteria and their corresponding phages (Table 2). The bacterial strains were cultured at 37°C in brain heart infusion (BHI) broth, providing suitable conditions for growth. The phages were routinely propagated on their host bacteria at 37°C.

Sodium dichloroisocyanurate (0.05% wt/vol, Chengdu Zhongguang Detergent Co., Ltd., China) (19), benzalkonium chloride (0.5% wt/vol, Shanghai, Jizhi Biochemical Technology Co., Ltd., China) (20), hydrogen peroxide (3% wt/vol, Chongqing, Ball Biotechnology Co., Ltd., China) (22, 90), and chlorhexidine gluconate (0.5% wt/vol, Chongqing, Shengbo biological reagent business department, China) (21, 22) were prepared using sterile water (autoclaved distilled water) and passed through a 0.45-µm filter membrane(Shanghai, Bioengineering Co., Ltd., China. The concentrations of the chemical disinfectants used in the experiments were prepared according to the manufacturers’ references (Table 1).

### Preparation of phages

Phages were prepared as previously described (91). First, 10 µL of the phage was added to 3 mL of the logarithmic phase bacteria and then cultured in a shaking incubator at 37°C for 4 hours. Following incubation, the mixture was centrifuged at 21,000 × g for 1 minute, and the supernatant was filtered through a 0.45-µm filter and stored at 4°C.

Phage titers were determined by the double-layer agar (DLA) method (92). Ten microliters of the serial 10-fold diluted phage solution was mixed with host bacteria (100 µL) and BHI soft agar (5 mL), which was then poured onto a BHI agar plate and incubated overnight at 37°C to observe plaque formation.

### The impact of chemical disinfectants on phages

To evaluate the sensitivity of the eight phages to different chemical disinfectants, several groups were established: the phage control group (phages plus sterile water), the chemical disinfectant control group (chemical disinfectant plus sterile water), and the experimental group (phages with chemical disinfectant). The samples from the experimental and control groups were incubated at 37°C for 1 hour. After incubation, the surviving phages in the samples were measured by DLA (92). The phage titer obtained in the experimental group was compared with that obtained in the phage control group to determine the survival rate of the phages.

### Biofilm removal experiment

The biofilm removal experiment commenced with biofilm preparation. An overnight bacterial culture was diluted in the BHI medium to achieve an optical density at 600 nm (OD600) 0.2. Subsequently, diluted bacterial suspensions were aliquoted and transferred to each well of a 96-well microtitration plate, where the bacterial volume was 200 µL per well. The plate was then incubated at 37°C for 24 hours under static conditions, allowing the formation of a biofilm (93, 94). Following the incubation period, the planktonic phase was carefully removed from the wells, and the biofilm was washed twice with sterile water (200 µL). Finally, the biofilm was left to dry at room temperature. In the biofilm removal experiment, a single phage, sodium dichloroisocyanurate (0.05% wt/vol), or a combination of phage and chemical disinfectant (200 µL) was added to the wells of the 96-well plate. The plate was then incubated at 37°C for 4 hours (69). Following the treatment period, the liquid was carefully removed, and the biofilm was washed twice with sterile water (200 µL). Subsequently, the biofilm was stained with 0.1% crystal violet (200 µL) for 20 minutes. After removing the excess stain, the biofilm was washed twice with sterile water and left to dry at room temperature for 3 hours. To quantify the biomass, 200 µL of 95% ethanol was added to each well for decolorization, and the optical density at 570 nm (OD570) was measured (69, 95).

To investigate the impact of sequential application of phages and chemical disinfectants on biofilm removal, two experiments were conducted. In Experiment 1, phages were applied for 4 hours followed by chemical disinfectants for another 4 hours. In Experiment 2, chemical disinfectants were applied for 4 hours followed by application of phages for another 4 hours. The subsequent assessment of biofilm removal effectiveness was performed as described previously. The control group received the BHI medium only. To ensure consistency in the culture system, the chemical disinfectant was diluted in the BHI liquid medium. The biofilm removal rate was calculated by comparing the biomass in the treatment groups to that in the control group, expressed as a percentage.

### Disinfection experiments of hard surfaces by phage and sodium dichloroisocyanurate

To evaluate the efficacy of phages and chemical disinfectants in disinfecting hard surfaces contaminated with pathogens, this study focused on three commonly encountered inanimate hard surface materials in clinical environments: plastic test tube caps (e.g., light switches and toilets), glass slides (e.g., windows), and stainless steel (e.g., door handles). These materials were first disinfected with 75% alcohol and then sterilized by autoclaving in steam for 30 minutes.

The first step involved contaminating the hard surfaces. In summary, the bacterial solution containing 10^10^ CFU was evenly distributed onto the tested surface (all ca. 25 × 25 mm in size) and spread using a pipette tip. The mixture was dried naturally and incubated at 37°C for 1 hour. The contaminated hard surfaces were subsequently divided into four groups for treatment: the first group served as the control and was treated with the BHI medium alone, the second group was treated with sodium dichloroisocyanurate (0.05% wt/vol) as the disinfectant experimental group, the third group was treated with phages as the phage experimental group, and the fourth group was treated with a combination of phages and sodium dichloroisocyanurate (0.05% wt/vol) as the combination experimental group. All treatment groups were kept at room temperature for 2 hours (96, 97). Following the treatment period, the treated hard surfaces were transferred to a centrifuge tube containing Dey and Engley (D/E) neutralizing broth. The surfaces were incubated in the broth for 10 minutes to ensure complete neutralization of any residual disinfectant and to facilitate the transfer of bacteria from the hard surfaces to the neutralizing broth. Subsequently, the number of bacteria in the neutralizing broth was determined by performing plate colony counting. This allowed for a comparison of the bacterial counts between the control group and the experimental groups (98).

### Data analysis

All experiments were conducted with a minimum of three biological replicates to ensure the reliability of the results. The experimental data were subjected to logarithmic transformation and analyzed using SPSS version 18.0 (SPSS Inc., Chicago, Ill., USA). Graphs were created using R version 4.1.3 (Bell Laboratories, Madison, WI, USA) with the ggplot2 package, utilizing the mean and standard deviations of the data sets (99). The laboratory examination indexes between the two groups were analyzed with the independent *t*-test, and the Bonferroni correction was applied to adjust *P*-values. Multiple group mean comparisons were performed with one-way analysis of variance (ANOVA) and Tukey test for post hoc comparisons. All tests were bilateral, and *P* < 0.05 was considered statistically significant.

## SUPPLEMENTAL MATERIAL

Table S1 spectrum.03797-23-s0001.docx Statistical analysis of Phage and chemical disinfectant remove biofilms efficiently.

Table S2 spectrum.03797-23-s0002.docx Statistical analysis of surface decontamination by the phages and sodium dichloroisocyanurate.

ASM does not own the copyrights to Supplemental Material that may be linked to, or accessed through, an article. The authors have granted ASM a non-exclusive, world-wide license to publish the Supplemental Material files. Please contact the corresponding author directly for reuse.
