# Supplementary material for: Effective elimination of bacteria on hard surfaces by the combined use of bacteriophages and chemical disinfectants
Source: Microbiol Spectr. 2024 Mar 14;12(4):e03797-23. doi: 10.1128/spectrum.03797-23 (PMC10986474; doi:10.1128/spectrum.03797-23)
Supplement: Table S1 — Statistical analysis of Phage and chemical disinfectant remove biofilms efficiently. [file spectrum.03797-23-s0001.docx]

**Table S1.** **Statistical analysis of Phage and chemical disinfectant remove biofilms** **efficiently.**

| Bacteria | Group | Mean±SD | P |
| --- | --- | --- | --- |
| JM110 | BHI | 0.891±0.219 | 0.018 |
|  | Disinfectant | 0.501±0.096 |  |
|  | BHI | 0.891±0.219 | <0.001 |
|  | Phage | 0.210±0.043 |  |
|  | BHI | 0.891±0.219 | 0.001 |
|  | Combination | 0.336±0.150 |  |
|  | Phage first | 0.192±0.058 | 0.971 |
|  | Disinfectant first | 0.266±0.036 |  |
|  | BHI | 0.891±0.219 | <0.001 |
|  | Phage first | 0.192±0.058 |  |
|  | BHI | 0.891±0.219 | <0.001 |
|  | Disinfectant first | 0.266±0.036 |  |
| THR60 | BHI | 2.516±0.758 | 0.243 |
|  | Disinfectant | 1.670±0.478 |  |
|  | BHI | 2.516±0.758 | 0.005 |
|  | Phage | 0.805±0.200 |  |
|  | BHI | 2.516±0.758 | 0.007 |
|  | Combination | 0.880±0.389 |  |
| PAO1 | Phage first | 0.281±0.011 | 0.009 |
|  | Disinfectant first | 0.641±0.122 |  |
|  | BHI | 1.373±0.182 | <0.001 |
|  | Phage first | 0.281±0.011 |  |
|  | BHI | 1.373±0.182 | <0.001 |
|  | Disinfectant first | 0.641±0.122 |  |
| Ab9 | Phage first | 0.081±0.004 | 0.097 |
|  | Disinfectant first | 0.287±0.036 |  |
|  | BHI | 0.762±0.154 | <0.001 |
|  | Phage first | 0.081±0.004 |  |
|  | BHI | 0.762±0.154 | <0.001 |
|  | Disinfectant first | 0.287±0.036 |  |
