# Supplementary material for: Effective elimination of bacteria on hard surfaces by the combined use of bacteriophages and chemical disinfectants
Source: Microbiol Spectr. 2024 Mar 14;12(4):e03797-23. doi: 10.1128/spectrum.03797-23 (PMC10986474; doi:10.1128/spectrum.03797-23)
Supplement: Table S2 — Statistical analysis of surface decontamination by the phages and sodium dichloroisocyanurate. [file spectrum.03797-23-s0002.docx]

**Table S2. Statistical analysis of surface decontamination by the phages and sodium dichloroisocyanurate.**

| Bacteria | Hard surface | Group | Mean±SD | P |
| --- | --- | --- | --- | --- |
| Ab9 | Plastic | Phage | 4.414±0.624 | <0.001 |
|  |  | Disinfectant | 6.952±0.048 |  |
|  |  | Combination | 4.748±0.084 | <0.001 |
|  |  | Disinfectant | 6.952±0.048 |  |
|  | Stainless steel | Phage | 3.693±0.088 | 0.476 |
|  |  | Disinfectant | 3.360±0.318 |  |
|  |  | Combination | 2.884±0.033 | 0.216 |
|  |  | Disinfectant | 3.360±0.318 |  |
| PAO1 | Glass | Phage | 0.000±0.000 | <0.001 |
|  |  | Disinfectant | 4.816±0.105 |  |
|  |  | Combination | 0.000±0.000 | <0.001 |
|  |  | Disinfectant | 4.816±0.105 |  |
|  | Stainless steel | Phage | 2.854±0.046 | <0.001 |
|  |  | Disinfectant | 3.973±0.122 |  |
|  |  | Combination | 0.000±0.000 | <0.001 |
|  |  | Disinfectant | 3.973±0.122 |  |
